# In this month’s Bulletin

**DOI:** 10.2471/BLT.18.000918

**Published:** 2018-09-01

In the editorial section, Amina J Mohammed and Tedros Adhanom Ghebreyesus (590) map the links between human health and sustainable development. Shambhu Acharya et al. (591) review the research published on this theme.

Shelly Chadha et al. (592) call for papers on the topic of hearing loss. 

In the news section, Göran Tomson tells Fiona Fleck (595–596) how scientists can help governments make economic progress without damaging the environment or depleting natural resources. Sima Barmania (597–598) reports on attempts to design sustainable airports and to mitigate harms to users. 

## Austria, Czechia, Estonia, France, Georgia, Germany, Hungary, Kyrgyzstan, Latvia, Lithuania, the Republic of Moldova, Poland, Sweden, United Kingdom of Great Britain and Northern Ireland. 

### Measuring the cost of care

Jonathan Cylus et al. (599–609) compare methods for measuring catastrophic health expenditure. 

## Bangladesh, Bhutan, India, Maldives, Nepal, Sri Lanka, Thailand, Timor-Leste.

### Paying for care

Hui Wang et al. (610–620) analyse data on household health expenditures. 

## Chile, Colombia, Mexico

### Promoting health

Kira Fortune et al. (621–626) describe health promotion initiatives.

## Global

### Progressive realization of the right-to-health

Judith Bueno de Mesquita et al. (627–633) support the use of human right accountability reviews to monitor progress.

### The hidden costs of bad practices

Timothy Ken Mackey et al. (634–643) propose a framework for reducing corruption in the health sector. 

### Differential health outcomes for men and women

Mary Manandhar et al. (644–653) critique a view of gender that is limited to women.

### Leaving no one behind

Ahmad Reza Hosseinpoor et al. (654–659) discuss the quantification of health inequalities. 

### Working with people

Asiya Odugleh-Kolev & John Parrish-Sprowl (660–661) explain why community engagement is needed for universal health coverage.

### Countering antimicrobial resistance

Katie Clifford et al. (662–664) examine the implications of poor quality veterinary medicines.

